# A Smart Wristband Integrated with an IoT-Based Alarming System for Real-Time Sweat Alcohol Monitoring

**DOI:** 10.3390/s22176435

**Published:** 2022-08-26

**Authors:** Kodchakorn Khemtonglang, Nataphiya Chaiyaphet, Tinnakorn Kumsaen, Chanyamon Chaiyachati, Oranat Chuchuen

**Affiliations:** 1 Department of Chemical Engineering, Faculty of Engineering, Khon Kaen University, Khon Kaen 40002, Thailand; 2 Biomedical Engineering Program, Faculty of Engineering, Khon Kaen University, Khon Kaen 40002, Thailand; 3 Mekong Health Science Research Institute, Khon Kaen University, Khon Kaen 40002, Thailand

**Keywords:** noninvasive sweat alcohol monitoring, metal oxide (MOX) gas sensor, wearable sensor, Internet of Things (IoT)

## Abstract

Breathalyzer is a common approach to measuring blood alcohol concentration (BAC) levels of individuals suspected of drunk driving. Nevertheless, this device is relatively high-cost, inconvenient for people with limited breathing capacity, and risky for COVID-19 exposure. Here, we designed and developed a smart wristband integrating a real-time noninvasive sweat alcohol metal oxide (MOX) gas sensor with a Drunk Mate, an Internet of Thing (IoT)-based alarming system. A MOX sensor acquired transdermal alcohol concentration (TAC) which was converted to BAC and sent via the IoT network to the Blynk application platform on a smartphone, triggering alarming messages on LINE Notify. A user would receive an immediate alarming message when his BAC level reached an illegal alcohol concentration limit (BAC 50 mg%; TAC 0.70 mg/mL). The sensor readings showed a high linear correlation with TAC (R^2^ = 0.9815; limit of detection = 0.045 mg/mL) in the range of 0.10–1.05 mg/mL alcohol concentration in artificial sweat, achieving an accuracy of 94.66%. The sensor readings of ethanol in water were not statistically significantly different (*p* > 0.05) from the measurements in artificial sweat and other sweat-related solutions, suggesting that the device responded specifically to ethanol and was not affected by other electrolytes in the artificial sweat. Moreover, the device could continuously monitor TAC levels simulated in real-time in an artificial sweat testing system. With the integration of an IoT-based alarming system, the smart wristband developed from a commercial gas sensor presented here offers a promising low-cost MOX gas sensor monitoring technology for noninvasive and real-time sweat alcohol measurement and monitoring.

## 1. Introduction

According to the report from WHO [1] and International Transport Forum [2], 1.25 million people died from road traffic accidents and 21.95% of those accidents were alcohol-related fatalities, which interpreted that one person died approximately every two minutes from alcohol-related road accidents worldwide in 2010. Moreover, road traffic injury was predicted by WHO to become the fifth leading cause of death in 2030 with a death rate increase of 63.6% from 2004 [3]. Random breath testing at sobriety checkpoints can effectively reduce fatal and nonfatal injuries from alcohol-related accidents, but many locations only implement checkpoints during holidays for short-term effects [4].

Blood alcohol concentration (BAC) measurement is a gold standard technique used to determine the intoxication level of the body, which can be estimated from two methods. Although gas chromatography is a universally accepted method for providing direct BAC measurement from blood sample analysis, it is an inconvenient and time-consuming method that requires a professional technician to perform a laboratory operation. Other indirect measurement methods use breath or other body fluids such as interstitial fluid (ISF), sweat, saliva, and tears to determine the BAC level indirectly [5]. The breathalyzer indirectly estimates BAC from breath alcohol concentration (BrAC), another reliable parameter showing a high linear correlation with BAC [6]. Even though a breathalyzer can provide a reasonably sensitive BAC level of up to 90–95% [7], its air testing chamber requires a relatively large amount of a deep breath to operate the measurement, which could present an obstacle for patients with respiratory system and lung diseases [8]. Additionally, according to the new guidelines released by the Australian government, the drug and alcohol monitoring using a breathalyzer should be adequately monitored during the COVID-19 pandemic. Even though there is a single-use mouthpiece for an individual when using a breathalyzer, the improper usage of breathalyzer and mouthpieces increases the risk of exposure to COVID-19 for both test takers and administrators [9].

Sweat has increased attention as one of the alternative body fluids for noninvasive health monitoring [10]. Because of the high linear correlation between TAC and BAC [11], the relatively high sensitivity, specificity, and low cost, sweat was considered as one of the valuable metabolites for noninvasive BAC level estimation. The MOX sensor has been developed and utilized as a homemade, low-cost alcohol monitoring device [12,13,14]. An MOX commercial gas sensor, MICS5524, was shown to have the highest sensitivity for TAC monitoring [12]. When ethanol molecules are exposed to the air, the sensing material’s resistance of the MOX sensor changes as a function of the partial pressure of the existing gas, resulting in the flow of electrons in the sensing material [15]. However, the study of the specificity of ethanol detection and monitoring by MOX sensors in sweat is rarely investigated [12,13,14]. Moreover, the design and development for wireless alcohol sensing devices have not been well studied.

In this work, we developed a smart wristband created from a low-cost commercial MOX gas sensor integrated with a real-time alarming system for sweat alcohol monitoring. The sensitivity, specificity, and accuracy of the MOX gas sensor was first investigated and optimized in both ideal and artificial sweat settings. Then, the wireless sensing system was designed and fabricated, including a semiconductor alcohol gas sensor, microcontroller board, and rechargeable battery in a wearable wrist-worn 3D-printed enclosure. Additionally, we developed a Drunk Mate, an IoT-based platform, to transfer the BAC converted from the measured TAC values to the Blynk application platform, triggering alarming messages on LINE Notify when the alcohol concentration reached particular levels. The alarming range covered TAC levels from the first drink to an illegal limit (BAC 50 mg%).

## 2. Materials and Methods

### 2.1. Chemicals and Materials

Ethanol (Ethyl alcohol 99.8%) was purchased from Merck & Co. Deionized water was purchased from RCI Labscan Limited (Bangkok, Thailand). Sodium chloride (NaCl), potassium chloride (KCl), lactic acid (C_3_H_6_O_3_), and urea (CH_4_N_2_O) were purchased from Sigma-Aldrich. Acetone (C_3_H_6_O) was purchased from GNS Chemical Co., Ltd (Songkhla, Thailand). All reagents were analytical grade and used without further purification.

The alcohol MOX gas sensor (MICS5524) was purchased from SGX Sensortech (Shanghai, China). The microcontroller (ESP32 WROOM-32 WiFi + Bluetooth development board) was purchased from Espressif Systems Co., Ltd. (Shanghai, China). Rechargeable lithium polymer battery 3.7 V 1000 mAh was purchased from Kayo Battery Co., Ltd. (Shenzhen, China). A 5 V 1 A booster and charger power bank module was purchased from Cytron Technologies (Bangkok, Thailand). A 3D printer (Anet A8 print) was purchased from Anet Technology Co., Ltd. (Shenzhen, China). Polyethylene terephthalate glycol-modified (PETG) filament was purchased from X3D Technology Palawatr Co., Ltd. (Nakhon Pathom, Thailand). Semi-permeable membrane (Opsite Flexifix©) was purchased from Smith & Nephew Ltd. (London, UK).

### 2.2. Artificial Sweat Formulation

In this work, artificial sweat was prepared from four main chemicals found in sweat according to the European standard number EN1811: 2012 by dissolving 0.5% *w/v* sodium chloride (NaCl), 0.1% *w/v* potassium chloride (KCl), 0.1% *w/v* lactic acid (C_3_H_6_O_3_), and 0.1% *w/v* urea (CH_4_N_2_O) into deionized water. For the sensor’s specificity analysis, acetone was evaluated at a concentration of 87 ppb because this is the physiological sweat acetone concentration reported in healthy individuals [16].

### 2.3. The Device Enclosure and System Design

#### 2.3.1. Device Enclosure Design

**Figure 1:**
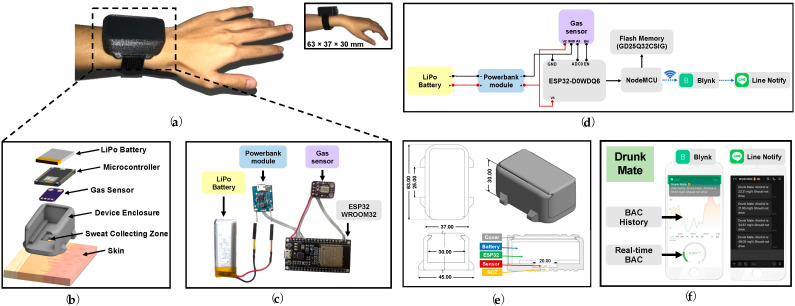
Schematic illustration of the smart wristband for real-time alcohol monitoring. (**a**) The smart wristband being worn on a wrist for illustration; (**b**) Design layout of the sensing system with device enclosure; (**c**) Components used in the device electronic system; (**d**) A schematic diagram of the sensing system, data acquisition, and the IoT-based alarming system; (**e**) The isometric and cross-sectional views with a dimension of the device enclosure; (**f**) The user interfaces of Drunk Mate, consisting of Blynk IoT platform and the LINE Notify messaging platform for real-time alarming notification.

An enclosure was designed to enable the sensing system, signal reader, IoT alarming system, and power supply to work efficiently together in a compact wrist-worn size, as shown in Figure 1a. The enclosure (Figure 1b) consisted of: (i) a 213.75 mm^3^ sweat collecting zone to confine a closed chamber for target gas capturing while keeping the sensor’s surface away from direct skin contact; (ii) the internal space for storage of ESP32, MOX gas sensor, power bank module, and LiPo battery (Figure 1c); and (iii) the sliding cover and watch bands attachment area for wrist-worn application.

#### 2.3.2. Alcohol Sensing and IoT-Based Alarming System Design

Figure 1d illustrates the overview of the system design. The lithium polymer rechargeable battery (LiPo battery) was connected to the power bank module which stepped up the voltage to 5 V to supply the ESP32 microcontroller board. The sweat ethanol concentration was detected by MICS5524 gas sensor, which operated at a surface temperature of 36.78 ± 0.31 °C, and processed by ESP32 then wirelessly sent by NodeMCU to the Drunk Mate (Figure 1f), consisting of the Blynk (Blynk, New York, NY, USA) application platform, which finally triggered alarming notifications on LINE Notify (LINE, Tokyo, Japan) application to user’s smartphone.

#### 2.3.3. A 3D Printing for Wristband Construction

The layout of the device was designed by Autodesk Inventor Software (version 2021.2.1, Autodesk, CA, USA). The device enclosure was designed to be attached to the skin around the user’s wrist similar to a regular watch. The enclosure was designed to use every space effectively with the size of 37 mm × 63 mm × 30 mm (displayed in an inset of Figure 1a).

The 3D-printer and Ultimaker Cura software (version 4.12, Ultimaker, Utrecht, The Netherlands) were used to construct the device (Autodesk layout displayed in Figure 1e) from the PETG filament and then attached to the commercial nylon watch bands.

### 2.4. Preparation of Experimental Testing Systems

#### 2.4.1. Calibration Testing System

The actual ethanol solutions (0.10–1.05 mg/mL) were prepared by serially diluting ethanol solution (as received) with deionized water and kept in a small fully-sealed jar. The ethanol concentration range in this study was chosen to cover the range of expected physiological sweat ethanol concentration. To create a calibration curve, the sensor was first connected to the data acquisition system (PLX-DAQ) for 30 s to stabilize the baseline readings before being connected to the sealed experimental jar for another 970 s. In total, the signal outputs were recorded three times (1000 s each time). The measured liquid alcohol concentrations were calculated by averaging 100 readings when the equilibrium state had been reached. The limit of detection (LOD) was calculated as 3.3 times the standard deviation of the blank (*n* = 10), divided by the slope of the calibration curve.

#### 2.4.2. Artificial Sweat Generating System

**Figure 2:**
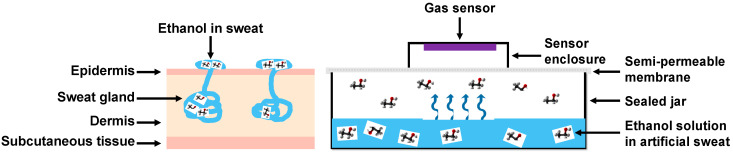
The illustration of an artificial sweat generating system (**right**) mimicking the human sweating system (**left**).

The sweat generating system developed by Sim et al. [17] was implemented to test the device’s ability in detecting transdermal alcohol concentration through a semi-permeable membrane, which was used by the authors to mimic an artificial skin layer. Figure 2 shows the functional similarity comparison between the human sweating pathway and the artificial sweat generating system. In the human sweating pathway, sweat generating from an eccrine gland is transported to the sweat duct, which goes through the epidermis layer and skin pores, respectively (shown in Figure 2 left). In the artificial sweat generating system, ethanol molecules dissolved in artificial sweat in the jar were transported to a layer of semi-permeable skin-like membrane (OPSITE Flexifix©). Next, the sweat was generated and evaporated through the membrane to its surface area (shown in Figure 2 right). The 3D-printed sensor enclosure connected with the alcohol gas sensor was placed on the membrane surface to record measurements. The experiment was run at room temperature (25 ± 5 °C).

#### 2.4.3. Specificity Testing Methodology

A series of experiments were conducted in an artificial sweat generating system to evaluate the sensor’s specificity to ethanol in nine testing solutions: deionized water (DI), artificial sweat (AS), acetone (87 ppb) in AS, ethanol (0.42 mg/mL) in AS, ethanol in acetone solution, ethanol in KCl solution, ethanol in lactic acid solution, ethanol in NaCl solution, and ethanol in urea solution. Each testing solution was already prepared in a jar covered with a semi-permeable membrane. The sensor readings were recorded 50 s in ambient air for baseline collection and then were recorded three times (2 min each time) on the semi-permeable membrane for ethanol concentration collection. For specificity analysis, the ethanol concentration readings were subtracted by the collected baseline to calculate the readings change from ethanol concentration only. Then the calculated data from 9 solutions were statistically evaluated by *t*-test analysis to validate the influences of artificial sweat and other sweat-related compounds in affecting the readings of the device.

#### 2.4.4. Accuracy Testing Methodology

The signal output of the gas sensor was investigated in an artificial sweat generating system in a blind test with six unknown alcohol samples. Each unknown was injected into the testing jar, and the signal output was recorded three times (5 min each time). The signal of each unknown sample was selected for 100 points for further calculation when the system had reached its stable state. Then, the signal was converted to TAC level using the calibration curve and then was calculated to obtain the error percentages.

### 2.5. Preparation of Real-Time Alcohol Monitoring and an IoT-Based Alarming System Analysis

#### 2.5.1. Real-Time Alcohol Monitoring

The real-time alcohol monitoring was evaluated by injecting an appropriate alcohol amount into the solution sink of the artificial sweat generating system to simulate the BAC level in a male (BMI of 28.0) after 1–4 standard drinks, calculated using the Widmark formula. The experiment was tested with two different setups. First, the non-continuous alcohol consumption setup evaluated the ability of the sensor to monitor one standard drink and two standard drinks separately. Each of the standard drink solutions was added separately to the ethanol sink of the testing system, and the signal output was recorded for over 50 min. Second, the continuous alcohol consumption setup evaluated the ability of the sensor to monitor the consumption of one standard drink, followed by another drink, continuously. Each standard drink solution was added into the testing system 15 min after each other for five drinks in total. This continuous alcohol consumption setup was designed to represent binge drinking behavior. A Drunk Mate system was set up to evaluate the alarming performance of the device along with this experiment.

#### 2.5.2. An IoT-Based Alarming System Analysis

The ESP32 was programmed to transmit the signal output from the alcohol gas sensor through an IoT platform to the Blynk application. The interface consisted of a real-time alcohol concentration gauge and an alcohol concentration history graph (Figure 1f left). For alarming notification, LINE Notify was programmed to categorize the calculated BAC result into three alarming levels and send notifications to the user in real-time (Figure 1f right). Three alarming messages were programmed to display according to the detected alcohol concentration levels based on Thailand’s drunk driving laws, illegal to drive at BAC level above 50 mg%: Level 1: OK to drive (BAC 0–20 mg%), level 2: Should not drive (BAC 20–50 mg%), and level 3: DRUNK: Do not drive (BAC 50 mg% above).

## 3. Results and Discussion

### 3.1. Sensitivity Optimization of Sweat Alcohol Monitoring Using MICS5524 MOX Gas Sensor

The experimental setup for testing the device’s sensitivity was conducted according to Henry’s law gas solubility in liquid. It was prepared in a small, fully sealed jar with a sink of ethanol solution to create an equilibrium state between ethanol in the liquid and gas phases. The actual samples of ethanol solution in artificial sweat (0.10–1.05 mg/mL) were tested with the MICS5524 MOX alcohol sensor in a sealed experimental jar for 1000 s until the system reached the liquid-gas equilibrium and sensor readings stabilized. 

**Figure 3:**
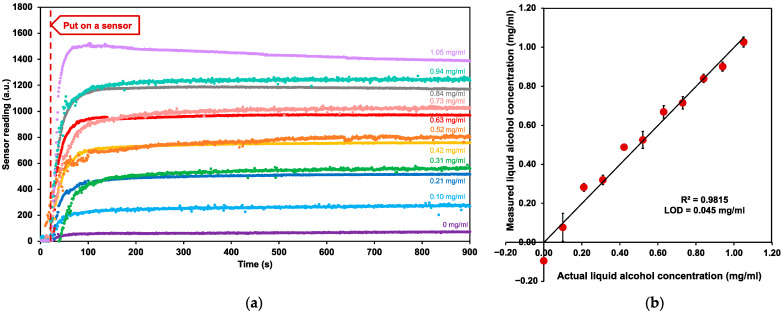
The sensitivity analysis (**a**) Sensor reading outputs in various ethanol concentrations in the range of 0.10–1.05 mg/mL recorded from the beginning until the system reached an equilibrium state (**b**) Correlation between the measured alcohol concentration versus the actual alcohol concentration in artificial sweat. Each data point represents mean ± standard deviation (*n* = 3).

Figure 3a shows the sensor reading of ten ethanol samples and one blank control sample that were recorded from the MICS5524 and transmitted through the ESP32. This result indicated that after putting the MICS5524 sensor onto the calibration testing system, the sensor readings immediately increased and stabilized after reaching its equilibrium state. As shown in Figure 3b, there was a strong linear correlation between the measured and actual liquid alcohol concentrations (R^2^ = 0.9815), with an LOD of 0.045 mg/mL. The linear correlation confirmed the ability of the MICS5524 sensor in monitoring sweat alcohol concentration in real-life application, covering the sweat alcohol range found in human after the first consumption of alcohol standard drink. The LOD ensured the ability in measuring a relatively low alcohol concentration using our optimized method and testing system.

### 3.2. Alcohol Specificity Analysis by Artificial Sweat Generating System

Sweat contains several analytes such as ions, metabolites, acids, hormones, and small proteins and peptides [10] that could possibly interfere with signal reading. This experiment evaluated how the signal interferences caused by those sweat components affected the ability of the MICS5524 sensor to detect alcohol. Acetone is a volatile organic compound (VOC) which can normally be found in the sweat of healthy persons and may indicate certain health-related problems if present at a relatively high level. The averaged acetone concentration levels in the sweat of healthy individuals and diabetes patients were reported as 87 ± 10 ppb and 188 ± 17 ppb, respectively [16]. Therefore, the sensor was also tested with an 87 ppb acetone solution in artificial sweat, which was the physiological sweat concentration level of acetone, in order to simulate its effects on the sensor’s selectivity performance.

**Figure 4:**
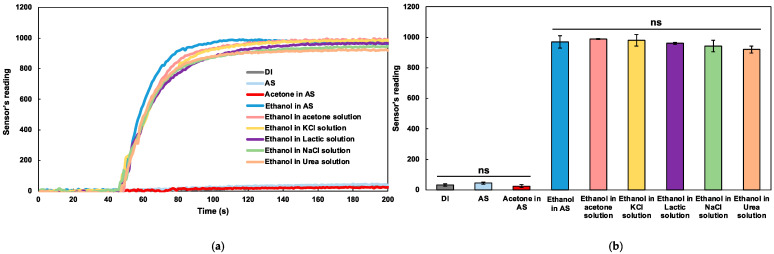
The alcohol specificity analysis (**a**) Raw sensor reading output of DI, AS, 87 ppb acetone in AS, 0.42 mg/mL ethanol in artificial sweat, 87 ppb acetone, KCl, lactic acid, NaCl, and urea solutions. (**b**) Processed sensor output with t-test analysis, suggesting that the device specifically responded to ethanol only. “ns” means no significant difference (*p* > 0.05). Data are presented as mean and standard deviation (*n* = 3).

The raw signal data and mean values of the signal outputs acquired from the MICS5524 sensor for nine different testing solutions are displayed in Figure 4a,b, respectively. The signal outputs from the five ethanol solutions in each sweat component (acetone, KCl, NaCl, lactic acid, and urea) did not differ significantly, but were significantly higher than the three control samples without ethanol (DI, AS, and acetone in AS). This finding revealed that the only component that contributed to the change of the signal was ethanol, whilst the sweat components did not significantly interfere with the signal output. Moreover, the signal response obtained from the acetone solution in artificial sweat was not statistically different from that of DI water and the blank artificial sweat. This result suggested that the presence of acetone at the physiological concentration level found in the sweat of healthy individuals did not significantly affect the sensor’s specificity performance in measuring alcohol. However, it is recommended that for future on-body measurements, the sensor baseline should be established for each individual due to potential intra-person variation in sweat acetone concentration level.

### 3.3. Device Accuracy Evaluation

**Figure 5:**
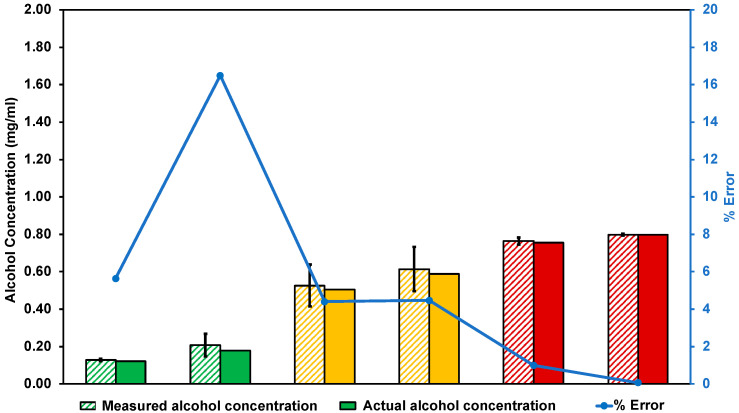
The comparison of measured (diagonal strip bars) and actual alcohol concentration (solid fill bars) from unknown samples with error percentages for sensor accuracy analysis. Data are presented as mean and standard deviation (*n* = 3).

Six unknown alcohol samples from the range of 0.10–1.05 mg/mL were used to evaluate the accuracy of the device in monitor sweat alcohol concentration. Each sample was recorded three times and the signal output was converted by the previously established calibration curve (Figure 3b) into concentration values. Figure 5 showed that the higher the alcohol concentration, the lower the error percentage. This device gave more than 97.5% accuracy at the concentration of 0.50 mg/mL and above, which could be considered as a reliable accuracy, covering the TAC level from a lower level to the illegal limit level (BAC 50 mg% = TAC 0.70 mg/mL). The green, yellow, and red colors in Figure 5 represented the TAC notification level 1, 2, and 3, respectively. The measured alcohol concentrations, a programmed parameter to trigger the alarming notifications, were relatively higher than the actual alcohol concentration. Therefore, this could be a benefit for the user to be warned of their BAC level in advance.

### 3.4. Real-Time Sweat Alcohol Monitoring and Evaluation of a Drunk Mate, IoT-Based System

**Figure 6:**
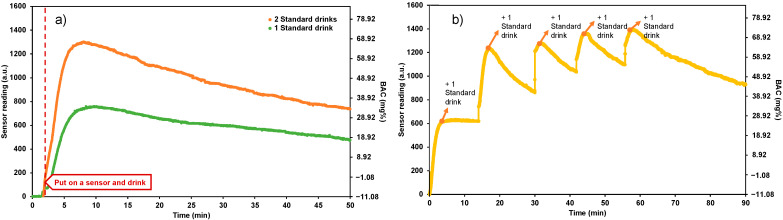
Result of real-time sweat alcohol monitoring in the artificial sweat generating system in two drinking behaviors (**a**) A no time-gap behavior from one drink and two drinks (**b**) A 15-min interval drinking behavior in multiple drinks.

The real-time alcohol monitoring experiment was conducted in the artificial sweat generating system designed to demonstrate the functional performance of the device in monitoring and warning the user when their BAC level has reached an illegal limit. Two drinking behaviors were simulated: a no time-gap drinking behavior (Figure 6a) and a 15-min interval drinking behavior (Figure 6b). The BAC level after alcohol consumption simulated in this experiment was calculated from the Widmark factor from Forrest [18] which takes into account the human alcohol metabolism and elimination rate, assuming that the subject was a male (BMI of 28.0) who consumed four standard drinks of alcohol beverage (14 grams of alcohol per one standard drink) for 0.5 h and had the alcohol elimination rate of 0.015 mg% per hour.

Figure 6a shows the sensor reading after putting the sensor on and applying the alcohol standard drink samples to simulate the BAC level for one and two standard drinks. Once the ethanol molecules reached the alcohol gas sensor, the sensor readings were immediately transmitted to Drunk Mate, which continuously displayed the user’s alcohol concentration on Blynk and sent out the warning messages on LINE Notify to inform a user of his BAC level in real-time. One alcohol standard drink had reached a BAC level of over 20 mg%, which triggered notifications in level 1 and 2. Similarly, since two alcohol standard drinks reached a BAC level of more than 50 mg%, it triggered notifications in levels 1, 2, and level 3, respectively.

In the experiment to simulate the 15-min interval drinking behavior (Figure 6b), one alcohol standard drink was applied to the ethanol sink of the artificial sweat generating system followed by the next drink applied with a 15-min time gap, and so on until a total of 5 standard drinks were applied within an hour. An increase in the BAC level was observed after the application of the first and second standard drink. After further applications, the BAC level increased in small increments. 

This experiment successfully demonstrated two sweat alcohol increasing patterns in the artificial sweat generating system and the monitoring and alarming capabilities of the Drunk Mate for over 1 h. It is important to note that the use of the calculated BAC from the Widmark formula and the sweat generating model adapted from Sim et al. (2016) was for proof-of-concept demonstration and validation only. This testing system did not fully and precisely replicate the entire perspiration and alcohol metabolism systems of the human body. Thus, evaluation of the sensor’s performance on human subjects should be the focus of future research.

### 3.5. Cost Estimation of the Smart Wristband

The total material and fabrication cost of this smart wristband device integrated with the IoT-based real-time alarming system was approximately $24.62 (Table 1). It was more than five times cheaper than commercial breathalyzers (BACtrack S80, AlcoMate Pro, and AlcoMate Core). Most of the components of this device are commercially available and relatively inexpensive. Thus, the device’s affordability and ease of fabrication may facilitate its mass-production potential.

## 4. Conclusions

In this work, a wristband for real-time sweat alcohol monitoring was designed and developed along with an IoT-based alarming system. First, the wristband enclosure that consisted of a sweat collecting zone and an electronic component compartment was designed and constructed using 3D-printing technology. Then, the MOX sensor’s sensitivity in measuring sweat alcohol concentration was studied. The device demonstrated a high linear correlation between sensor reading and TAC level (R^2^ = 0.9815) ranging from 0.10–1.05 mg/mL (LOD = 0.045 mg/mL), covering the expected sweat alcohol concentrations from the first drink to the illegal level (BAC 50 mg% and beyond) based on the drunk driving law in most European nations. The device integrated with Drunk Mate, an IoT-based alarming platform, could monitor the sweat alcohol concentration with 94.66% accuracy. The MOX alcohol sensor specifically responded to alcohol only without any influences of sweat components. The Drunk Mate platform gave a real-time alcohol concentration displayed on the Blynk application with three-level alarming messages on LINE Notify, showing the potential in reducing the chance of COVID-19 exposure during alcohol monitoring process. This study created a smart wristband from a low-cost commercial MOX sensor and investigated the sensitivity, specificity, and limit of detection of the device. It also presented the device’s performance for noninvasive and real-time sweat alcohol monitoring by integrating with wireless sensing and an IoT-based alarming system. In the next phase of this research, the capability and performance of the device for transdermal alcohol detection and monitoring in human will be evaluated.

## Figures and Tables

**Table 1:** Cost estimation of the smart wristband.

Components	Cost (USD)
MICS5524 MOX gas sensor	9.60
ESP32 Microcontroller	5.33
3.7 V LiPo Battery	3.82
Step-up module	0.85
Jumper wires	0.96
Device enclosure	4.05
Total	24.62

The cost was converted from THB when 1 USD = 35.30 THB.

## Data Availability

Not applicable.
